# Real-Time Type 1 Diabetes Self-Management Decision-Making in Adolescents: Protocol for a Longitudinal Mixed Methods Study Using Text Messaging and Continuous Glucose Monitoring

**DOI:** 10.2196/83218

**Published:** 2026-03-04

**Authors:** Melissa DeJonckheere, Samantha A Chuisano, Juniar Lucien, Fouzaan Amjad, Oorvi Duvvuri, Hasan Khan, Maryam Khan, Rafee Mirza, Maya Joy Ollivierre, Timothy Guetterman, Yu Kuei Lin, Lorraine R Buis, James E Aikens, Caroline Richardson, Joyce M Lee

**Affiliations:** 1 Institute for Healthcare Policy and Innovation, University of Michigan, Ann Arbor, MI, United States; 2 Department of Family Medicine, University of Michigan, 1018 Fuller Street, Ann Arbor, MI, 48104, United States, 1 7349361927; 3 Department of Internal Medicine, University of Michigan, Ann Arbor, MI, United States; 4 School of Information, University of Michigan, Ann Arbor, MI, United States; 5 Department of Pediatrics, University of Michigan, Ann Arbor, MI, United States; 6 Susan B. Meister Child Health Evaluation and Research Center, University of Michigan, Ann Arbor, MI, United States; 7 Elizabeth Weiser Caswell Diabetes Institute, University of Michigan, Ann Arbor, MI, United States

**Keywords:** type 1 diabetes, adolescent, youth, continuous glucose monitoring, CGM, diabetes, self-management, SMS text messaging, qualitative research, mixed methods

## Abstract

**Background:**

Type 1 diabetes (T1D) requires repeated self-management behaviors and ongoing problem-solving to maintain optimal glucose levels and prevent complications. Despite increasing adoption of continuous glucose monitoring (CGM), which can alleviate some of the constant self-management burden, adolescents struggle to achieve glycemic recommendations and report low engagement with diabetes device data. Previous studies have used retrospective or quantitative approaches to describe adolescent self-management; however, it is unclear how psychosocial influences (eg, mood and distress) and contexts impact adolescent self-management behaviors and engagement with their diabetes devices in everyday life. Exploration of real-time experiences will help to identify potential targets and strategies for future interventions to improve glycemic outcomes in adolescents with T1D using advanced diabetes technologies.

**Objective:**

This study has two aims: (1) to develop a grounded theory of self-management decision-making using diabetes devices among adolescents with T1D and (2) to assess the acceptability and feasibility of longitudinal and real-time qualitative data collection methods in this population.

**Methods:**

We will conduct a mixed methods study informed by the capability, opportunities, and motivation of behavior model. Adolescents (aged 12‐18 y) with T1D who regularly use CGMs will be recruited from a Midwest pediatric diabetes clinic. Purposive sampling strategy will ensure participants with varied glycemic levels (hemoglobin A_1c_ [HbA_1c_] ≤9% and HbA_1c_ >9%) and diabetes experiences (eg, diabetes duration, devices used) are included. Using a longitudinal convergent mixed methods design, enrolled participants (n=30‐40) will complete data collection over 6 weeks including: (1) a baseline survey to capture demographic, clinical, and behavioral characteristics; (2) 30 days of SMS text messaging surveys to describe real-time self-management behaviors, technology use, and decision-making; (3) 30 days of CGM data; and (4) an interview focused on self-management behaviors and technology use. Recruitment will continue until appropriate data completeness and/or theoretical saturation is achieved. Analysis of text responses and interview transcripts will follow a grounded theory approach. Summarized glycemic metrics (eg, time in range) and visuals (ie, ambulatory glucose profile) will be integrated with qualitative findings through participant profiles and joint displays. Integrated findings will be used to refine a grounded theory of daily self-management decision-making using diabetes devices among adolescents with T1D.

**Results:**

As of December 2025, 25 participants have enrolled in this study. We expect SMS text messaging survey completion rates and CGM use near 70% throughout the study period. We anticipate findings to become available in the following several years through conference presentations and peer-reviewed publications.

**Conclusions:**

While routine diabetes self-management behaviors and use of diabetes technologies are important for achieving glycemic goals, adolescents report low adherence to diabetes devices. This real-time mixed methods study will improve our understanding of daily decision-making and influences on diabetes self-management. Findings from this study will identify facilitators and barriers to optimal T1D self-management. In addition, results will inform future studies using real-time qualitative and mixed methods approaches.

## Introduction

Type 1 diabetes (T1D) is one of the most common chronic conditions among children globally, affecting 3.5 per 1000 youth in the United States [1,2]. T1D requires intensive daily management of blood glucose levels to reach hemoglobin A_1c_ (HbA_1c_) targets and minimize the risk of severe short- and long-term complications, including diabetic ketoacidosis, neuropathy, retinopathy, and cardiovascular disease [3]. In 2021, the American Diabetes Association recommended glycemic targets of less than 7% for most children and adolescents [4], yet only 19% of youth met this target in 2021 to 2023 [5]. As HbA_1c_ typically peaks in adolescence and young adulthood [6], it is a critical period to address barriers to self-management to reach optimal glycemic levels and prevent common complications that can persist through adulthood [7-9].

Elevated glycemic levels in adolescence result from the complex interplay of physical, emotional, and cognitive development in addition to social and structural determinants of health (eg, access to medical care and health care costs) [3,10-12]. Regular T1D self-management requires frequent blood glucose monitoring, regular insulin use, monitoring carbohydrate intake, modifying insulin use to match diet and activity levels, and troubleshooting unpredictable blood glucose levels. As a result, executive functioning (including planning, decision-making, problem-solving, and reflecting on blood glucose patterns and behaviors) plays a critical role in self-management [6,13,14]. As adolescents begin to take on increased responsibility for their own diabetes care and seek independence from their families, they often struggle to consistently perform these complex self-management behaviors [15,16]. Unsurprisingly, one-third of adolescents with T1D experience high levels of diabetes distress [17,18], which has been linked to poorer adherence to self-management and higher glycemic levels in adolescents [17,19].

Advanced diabetes technologies such as continuous glucose monitoring (CGM) may lessen the burden of diabetes self-management by providing real-time glucose values, alerting adolescents and caregivers to low and high blood glucose levels, and indicating the predicted direction of blood glucose levels in the next hour [20,21]. As of 2023, nearly 80% of adolescents with T1D use CGM [1]; however, the average HbA_1c_ among CGM users in this age group remains above the recommended level [22], suggesting that CGM alone is not enough for youth to meet HbA_1c_ targets.

While adolescents who regularly use CGM report high levels of satisfaction and quality of life [23], they also report concerns about device visibility, feeling “spied on,” alarm and device fatigue, feeling as though device data are unreliable or untrustworthy, and being overwhelmed by constant streams of data [24]. The emotional, social, and practical burden of CGM can contribute to distress, reduced motivation for device engagement, and, ultimately, lower device adherence [25]. Fewer than 30% of individuals with T1D report using any real-time CGM features such as event logging or data sharing, and less than 75% have customized their high and low alert thresholds [26]. While reviewing CGM data may support problem-solving and decision-making among adolescents with T1D [27,28], fewer than 20% of adolescents review their diabetes device data independently [23]. Additional research is needed to understand how adolescents are leveraging (or not leveraging) diabetes devices for self-management decisions in daily life, and how the use of diabetes devices impacts their overall health and well-being. Understanding how adolescents use their devices can help health care teams and families to identify key barriers to self-management and support young people to optimize self-management and care.

The overarching purpose of the Qualitative Insights to Type 1 Diabetes in Youth (QUALITY) study described here is to identify potential targets and strategies for future interventions to improve glycemic outcomes in adolescents with T1D using advanced diabetes technologies. The study has two aims: (1) to develop a grounded theory of self-management decision-making using diabetes devices among adolescents with T1D and (2) to assess the acceptability and feasibility of longitudinal and real-time qualitative data collection methods in this population. This observational study leverages a longitudinal, convergent mixed methods design, merging real-time narrative and CGM data to understand daily diabetes self-management behaviors, decision-making, and problem-solving among adolescents with T1D who use CGM. To our knowledge, this is the first application of a real-time and longitudinal mixed methods approach among adolescents with T1D. Thus, a secondary aim of the study is to assess the acceptability and feasibility of the proposed methods.

## Methods

### Overview

This longitudinal, convergent mixed methods study leverages a real-time approach to develop a theory of self-management among adolescents with T1D who use CGM. We will conduct a grounded theory analysis to describe self-management in this population, which will inform future intervention development. We aim to enroll approximately 30 to 40 adolescents with T1D who already use CGM to complete 30 days of real-time, narrative data collection via SMS text messaging and 30 days of CGM. Additional data will be collected via a baseline survey and one-time follow-up interview to further develop the theory.

When applied to diabetes, real-time data collection (eg, ecological momentary assessment) can allow us to more accurately assess T1D self-management beliefs, behaviors, and event experiences, reduce recall bias, help researchers to understand behaviors in context, and support the development of timely and tailored interventions [29-32]. With the growth of CGM among adolescents, continuous and real-time glucose data have been incorporated into momentary assessments [1,33] While real-time quantitative assessments help to characterize important variations that influence self-management, the broader context of an individual’s experience during those moments remains poorly understood. A mixed methods approach blending real-time and contextual data could address these gaps to provide a more comprehensive understanding of adolescents’ experiences and identify potential intervention targets and strategies. In this study, we will use a qualitative real-time approach to further explore the lived experience of T1D self-management from the perspectives of adolescents using diabetes technology.

### Conceptual Model

The QUALITY study is informed by the capability, opportunity, and motivation model of behavior (COM-B) [34] by assessing several relevant influences on adolescent self-management behaviors and exploring these behaviors through the routine, reactive, and reflective cycles that are performed in real-time by adolescents with T1D using diabetes devices. The COM-B model [34] serves as a helpful framework for describing T1D self-management behaviors in adolescents who use diabetes technology. According to the COM-B model, to perform a particular behavior, an individual must feel that they are capable of doing so (physically and psychologically), have the opportunity to do so (socially and physically), and be motivated to do so (automatically and reflectively) [34]. COM-B has frequently been used as a tool to develop intervention targets to change individual behaviors and can also be used as a framework to explore and describe influences on diabetes self-management [35-37]. For example, in the context of T1D self-management, the behavior of “checking blood glucose levels” would require the knowledge and skills needed to test through finger pricks or look at a CGM display (ie, capability), access to glucometers or CGM and comfort checking blood glucose levels in front of others (ie, opportunity), and the belief that testing blood glucose levels is desirable and impactful on health (ie, motivation).

**Figure 1.**
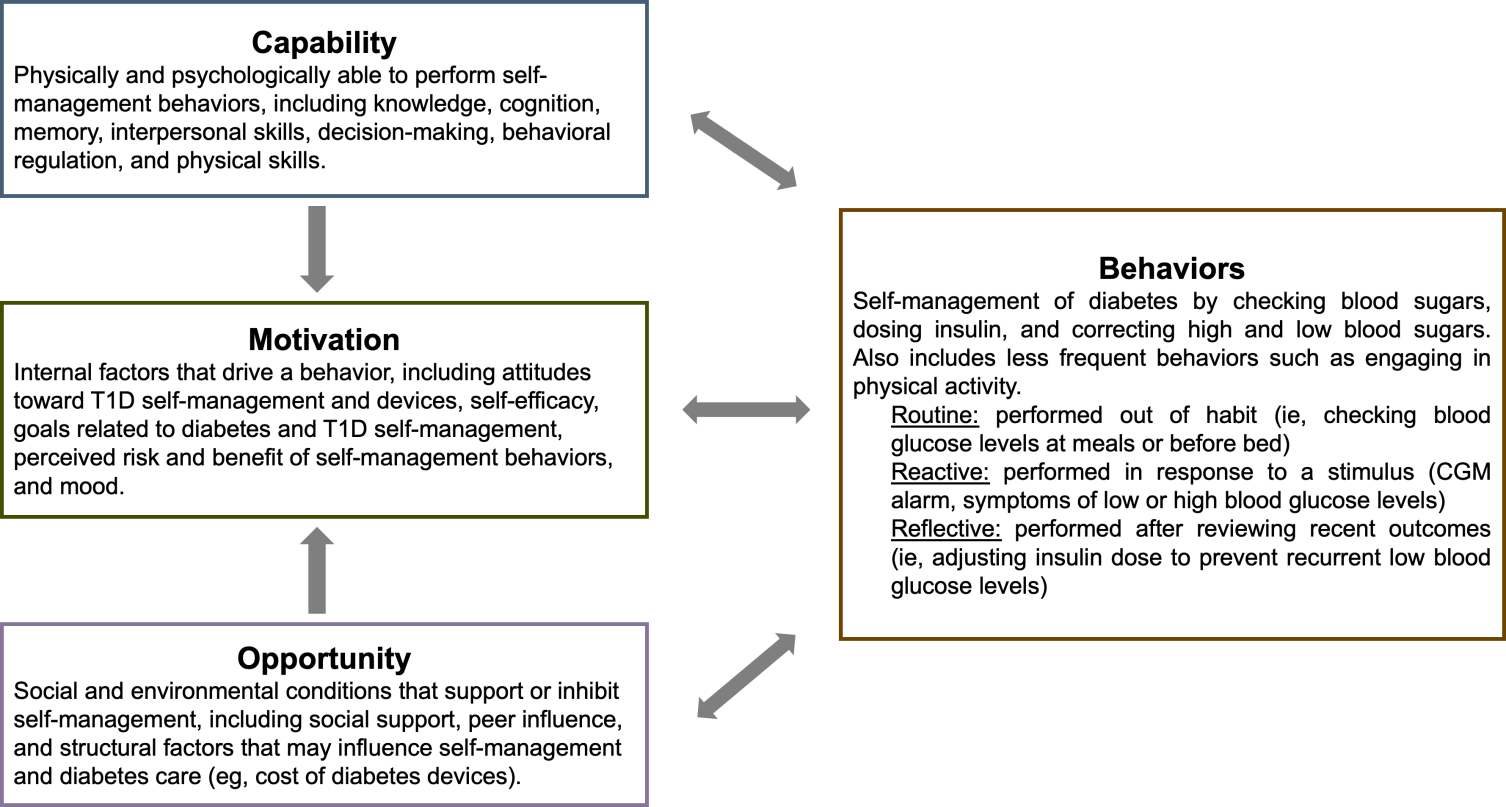
Capability, opportunity, and motivation of behavior (COM-B) conceptual model adapted for Qualitative Insights to Type 1 Diabetes in Youth (QUALITY) study. CGM: continuous glucose monitoring; T1D: type 1 diabetes.

T1D requires many interconnected behaviors to optimize self-management. Through an expert panel and literature review, Hamilton et al [38] identified 150 distinct self-management behaviors related to T1D that are performed in three self-regulatory behavioral cycles: (1) routine, (2) reactive, and (3) reflective. Each of these cycles is supported by or inhibited by capability, opportunities, and motivation for a given behavior [38]. Re-examining the example above of checking blood glucose levels, an adolescent with T1D may decide to check their glucose using CGM as a part of their daily self-management routine (ie, routine behavior), in response to hypoglycemia or hyperglycemia symptoms or a CGM alarm (ie, reactive behavior), or by reviewing trends in glucose available on their insulin pump, CGM receiver, or CGM mobile app to make later adjustments (ie, reflective behavior). COM-B highlights the complexity of improving self-management behaviors in adolescence, as each individual behavior is influenced by the interplay of capabilities, opportunities, and motivations in routine, reactive, and reflective contexts which occur every day, in real-time, and in diverse settings. Figure 1 presents the adapted COM-B used to inform the QUALITY study.

To ensure a youth-centered design and improve engagement with this study, the study team will make iterative changes to the study design and data collection tools based on feedback from an online patient advisory council. The Type 1 Diabetes Youth Advisory Council (T1DYAC) was established in 2024 to guide T1D research conducted primarily in our laboratory. T1DYAC members are youth (ages 14‐24 y) with T1D who have an interest in improving research about T1D in youth through discussion with research teams. T1DYAC members represent a range of ages, diabetes durations and histories, and diabetes devices used. During monthly virtual meetings, the group is presented with a research study and provides feedback on a specific study element (eg, recruitment flyers and approach, research questions, and interpretation of findings). In addition, members complete surveys between monthly meetings to provide written feedback and pointed insights on study materials.

### Setting

This study will recruit patients from the University of Michigan (U-M) Pediatric Diabetes Clinic at C.S. Mott Children’s Hospital. As of December 2025, there are approximately 700 current patients aged 12 to 18 years with T1D; among these, 357 (50%) patients are male, 554 (79%) are White, 85 (12%) are Black or African American, 12 (2%) are Asian, 36 (5%) are more than one or another race, and 36 (5%) are Hispanic or Latino. In addition, 417 of these patients (62%) have public insurance, and 687 (98%) are English speaking. Likewise, over 92% currently use CGM. The makeup of the clinical population is similar to that of the US population of people with T1D, where 68% are non-Hispanic White, 16% African American, 13% Hispanic, 3% other races, and nearly 80% use CGM [39]. While disparities persist, differences in CGM by age, race and ethnicity, and insurance type have narrowed significantly in the last 15 years [2,5].

### Recruitment and Sampling

A list of patients with T1D, aged 12 to 18 years, who (1) use CGM, (2) received care in the U-M Pediatric Diabetes Clinic in the past 12 months, and (3) have at least 1 HbA_1c_ laboratory result from the past 12 months will be generated using DataDirect (University of Michigan Medical School) and Electronic Medical Record Search Engine (EMERSE) [40]—university-developed tools for performing systematic searches of structured and unstructured patient medical records [41]. The list will include basic demographic, clinical, and contact information for identified patients.

Consistent with a grounded theory approach, we will use theoretical sampling to help ensure that adequate data are collected to address our research aims. For our initial sampling strategy (a priori) [42], we seek to enroll participants with diverse diabetes outcomes and experiences. We will intentionally recruit and enroll participants with HbA_1c_ of 9% or less and more than 9% to ensure adolescents with low and high glycemic levels are represented. We aim to recruit and enroll approximately equal groups with high and low glycemic levels. A purposive sampling approach will also be leveraged using demographic and clinical factors including age, gender, years since diagnosis, and other social determinants of health (eg, family income and food insecurity measures) to create a sample with varied experiences and backgrounds. Sociodemographic backgrounds that are under-represented within the clinical population (ie, Hispanic or Latino) in the sample will be intentionally oversampled. As data collection and analysis proceed, recruitment and sampling strategies may shift to explore variation in participant responses, develop relationships between codes and categories, and add details to the descriptions and characteristics of categories in the analysis [42].

Recruitment will be conducted in waves to allow for flexibility and needed changes to meet theoretical sampling goals. During each wave, approximately 100 to 200 identified patients will be contacted via electronic and/or postal mail up to 5 times with study information and a link to an online screening survey to confirm interest and eligibility. Study information will also be shared through (1) flyers and patient newsletters posted by the U-M Pediatric Diabetes Clinic, (2) UMHealthResearch.org, a secure platform at U-M for sharing study information with potential participants, and (3) emails and newsletters shared with patient diabetes councils. If recruitment goals are not reached through these methods, patients may also be approached in person at the U-M Pediatric Diabetes Clinic by nonclinician members of the study team.

Study eligibility criteria include: (1) current patient at Michigan Medicine with at least 1 visit and HbA_1c_ measurement in the last 6 months, (2) aged 12 to 18 years upon enrollment, (3) diagnosed with T1D for at least 6 months, (4) used CGM for at least 6 months, (5) used CGM for at least 20 days per month, (6) able to communicate in English, and (7) have access to a phone with SMS text messaging capabilities. Eligibility will be assessed via the screening survey and confirmed via medical record review.

A sample size estimate (N=30‐40) was determined a priori based on estimates of the amount of qualitative data needed to reach data sufficiency across participants with varied glycemic levels and diabetes experiences [43,44]. Due to the longitudinal nature of data collection and multiple data sources in this study, we anticipate having sufficient data to inform our grounded theory. As is common in qualitative research and grounded theory, additional data may be collected as needed to further develop findings and reach theoretical saturation [45]. The same sample will be used for quantitative data collection, including baseline surveys of diabetes characteristics and psychosocial measures, and 30 days of CGM data. Using identical samples in a convergent mixed methods design reduces threats to validity (eg, sample size legitimation) and ensures that appropriate conclusions can be drawn through integration [46,47].

### Ethical Considerations

All participants (and a parent or guardian for minors) will complete an enrollment call to review the informed consent document with a member of the study team and provide electronic or written consent or assent to enroll in the study. Throughout the study, survey invitations will include the study team’s contact information in the event of any questions or concerns. Participants will also receive contact information for the pediatric diabetes clinic and reminders to reach out to their diabetes care team or the provider on call with diabetes-related questions, concerns, and emergencies. In addition, participants will be instructed not to share any identifiable information through SMS text messaging (ie, name and school) as it is not a secure method of communication. All incoming survey responses will be stored securely, and the exported data will be deidentified for analysis. Participants can earn up to US $150 for completing all study activities, and final payments will be sent as a gift card or a check in the mail, depending on the participant’s preference. This study was reviewed and approved by the U-M Institutional Review Board (HUM00230299) prior to beginning recruitment.

### Data Collection Procedures

**Figure 2.**
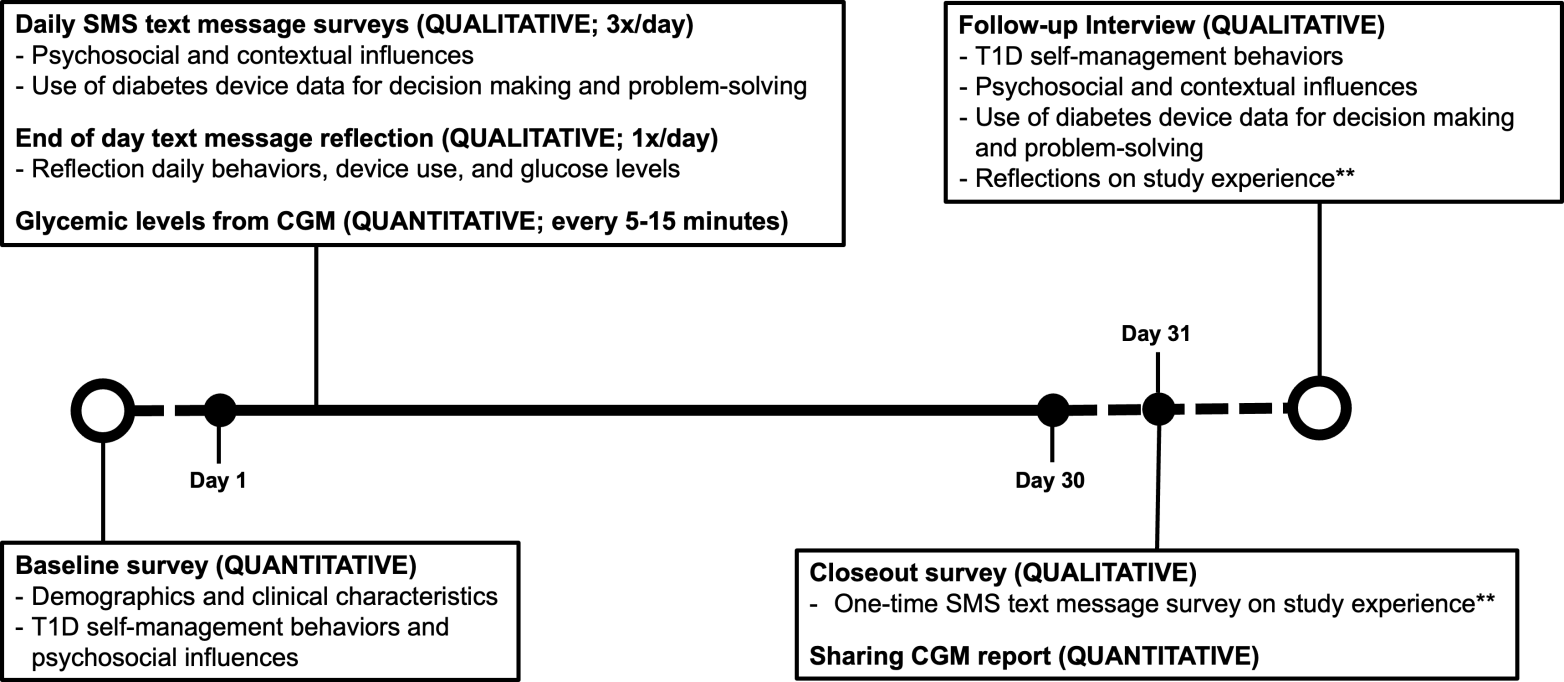
Qualitative Insights to Type 1 Diabetes in Youth (QUALITY) study participant flow. The asterisks indicate the data collection procedures related to study feasibility and acceptability. CGM: continuous glucose monitoring; T1D: type 1 diabetes.

Overall study participation will last approximately 6 weeks. Participants will complete (1) a one-time baseline survey to collect participant demographics, diabetes history, and psychosocial influences on T1D self-management; (2) 30 days of daily SMS text messaging surveys and CGM to evaluate real-time psychosocial influences on T1D self-management and glycemic levels; and (3) an optional semistructured interview to gain additional insights on T1D self-management approaches. Following enrollment and prior to data collection, participants will receive basic training on how to complete the daily surveys using a practice survey that includes sample questions with various formats (open-ended and closed-ended) and provides examples of survey prompts and outro messages. All survey data will be collected and managed using a secure REDCap (Research Electronic Data Capture; Vanderbilt University) database [48,49]. A study timeline and overview of data collection procedures for each participant are presented in Figure 2.

### Demographics and Clinical Characteristics

Participant demographic information and clinical characteristics will be collected upon enrollment using an online survey. Demographic measures include age, race and ethnicity, gender, and student and/or employment status. Clinical characteristics include age at diabetes diagnosis, recent (within the last 12 mo) hospitalization for diabetes-related complications, typical medical care for diabetes, and social support for diabetes management. Finally, questions on diabetes technology use include which type and brand of CGM and insulin pump are used, for how long, and typical use (how many d in the mo and use of automated insulin delivery). The participant demographics and clinical characteristics items are included in Multimedia Appendix 1.

### Baseline Survey

Study participants will complete a one-time baseline survey. Measures were selected to align with components of the COM-B model and the literature on psychosocial influences on self-management and diabetes device use. The survey includes measures of diabetes self-management behaviors, depression, diabetes distress, attitudes towards diabetes technology, problem-solving, and self-efficacy with diabetes self-management. A summary of survey instruments, sample items, and their alignment with the COM-B model is included in Table 1. Additional details can be found in Multimedia Appendix 2.

**Table 1.** Alignment of capability, opportunities, and motivation of behavior (COM-B) model with select data collection measures.

COM-B construct	Baseline survey items	Daily and end-of-day survey items	Semistructured interview questions
Capability (physically and psychologically able, including knowledge, decision-making, and skills)	Self-efficacy for T1D^a^ management: “[Can you…] adjust your insulin or food accurately based on how much exercise you get.” [50]. CGM^b^ self-efficacy: “I am sure I can... adjust insulin dose based on real-time data” [51].	Did you look at information from your diabetes devices in the last few hours? If no, why not?	What do you consider to be a high blood sugar? Why is this number significant to you?
Opportunity (social and environmental conditions, including social support and structural factors)	Clinical characteristics: For how long have you used CGM? How many days in the last month did you wear a CGM sensor or use CGM?	What problems, if any, did you encounter today? Were your diabetes devices useful to you today? How?	What support do you get from your health care providers that help you to use your technology?
Motivation (internal factors, including mood, attitudes, and beliefs)	Diabetes Technology Attitudes Scale: “Diabetes technology has made my life better” [52]. Benefits and burdens of CGM: “CGM alarms are helpful” and “CGM sensor readings cannot be trusted” [53]. Self-efficacy for T1D management: “[Taking care of my diabetes would] be too much responsibility” [50]. Problem areas in diabetes—teen: “Not feeling motivated to keep up with my daily diabetes tasks” [54]. Patient Health Questionnaire (2): “Feeling little interest or pleasure in doing things” [55].	How did you feel after looking at information from your diabetes device(s)? What else impacted how you took care of your diabetes today?	How confident do you feel making decisions in response to your current blood sugar?
Behavior			
Routine	Diabetes Management Questionnaire: “…adjust your food or insulin before long periods of physical activity?” [56]	Did you look at information from your diabetes devices in the last few hours? (If no) Why not?	Tell me about your general day-to-day approach to taking care of your diabetes.
Reactive	N/A^c^	What did you learn from your diabetes device(s)? What did you do next? Or how did you use the information?	When was the last time that you had a high blood sugar? Once you realized, what did you do next?
Reflective	Diabetes Adolescent Problem-Solving Questionnaire: “When I think a situation will get in the way of taking care of my diabetes, I do something to prevent that from happening” [57].	Use the daily graph in your CGM graph to review your blood sugars over the last 24 hours. Pick a time period you want to tell us more about (eg, 8 AM to 10 AM). What time did you pick and why? Walk us through what you think impacted your blood glucose levels during this time of day. Do you notice any patterns between your blood glucose levels in the past week? Tell us about it.	What do you do to try to make CGM more helpful to you?

a T1D: type 1 diabetes.

b CGM: continuous glucose monitoring.

c N/A: not applicable.

### Real-Time Daily Data Collection

SMS text messaging survey data will be collected using the “survey as a text conversation” feature in REDCap [48,49], which mimics a typical SMS text messaging conversation where questions are sent one-at-a-time and each question is sent only after a response is received. Surveys will automatically close after completion or after 3 hours if no response is received, and the next survey will automatically be sent at the prescheduled time. SMS text messaging was selected for daily surveys based on discussions with T1DYAC members, the high prevalence of smartphone use among US teens (95%) [58], and high rates of smartphone initiation by the age of 12 years (75%) [59]. In addition, the daily and end-of-day surveys were designed to have no more than 10 questions at a time to reduce the overall participant burden.

Beginning after the baseline survey is completed, participants will receive SMS text messaging survey invitations 4 times per day on a fixed schedule in the morning (10 AM), afternoon (2 PM), early evening (6 PM), and end-of-day (10 PM) [3] The morning, afternoon, and early evening surveys focus on interactions with their diabetes technology, challenges in managing diabetes, and other events that have occurred in the last few hours. For example, 1 question asks, “What have you been doing in the last few hours (ie, exercising, eating, at school, etc)?” In the evenings (10 PM), participants will complete a guided reflection using open-ended questions. During the reflection, they will be asked to open their diabetes device app and review the graph of their glucose levels throughout the day. Qualitative and quantitative data will be integrated during data collection as participants provide written reflections on their CGM data in daily SMS text messaging surveys. In addition, they will share information about what activities they did that day and how they feel events and activities impacted their diabetes. For example, the first question asks, “Overall, how did you feel about your diabetes today?” Sample daily and end-of-day survey question items and their alignment with the COM-B model can be found in Table 1. Complete daily and end-of-day SMS text messaging surveys are included in Multimedia Appendix 3. Participant response rates will be tracked automatically by REDCap throughout the study. In addition, participants will complete an open-ended text messaging survey after the 30 days of daily surveys are completed to qualitatively share their study experiences, burden, and study acceptability.

During the same 30 days, participants will be asked to continue CGM as prescribed to collect glucose levels. Depending on the device manufacturer, glucose levels are measured automatically every 1 to 15 minutes. By not introducing a new device, reactivity is reduced, and we are able to capture typical self-management behaviors and glycemic levels in this population. Likewise, participants do not have to learn to use a different device solely for the purpose of this study. Glycemic data are not blinded from participants to maintain normalcy with their daily routine and to maintain their safety in managing potentially dangerous high and low glucose levels during the study period. After completion of the daily survey portion of the study, participants will receive a secure REDCap survey link to share their CGM data from the previous 30 days downloaded as a .csv file from their CGM platform (eg, Dexcom Clarity). In addition, participants will be asked to reflect on the feasibility and acceptability of the study through 7 survey questions. Questions include study barriers, facilitators, and reflections on the length and timing of data collection (see Multimedia Appendix 4 for full closeout survey).

### Semistructured Interview

A subsample of participants (n=20‐30) will be invited to complete a 1-hour follow-up interview with a member of the study team trained in qualitative data collection and analysis. A maximum variation sampling approach will be leveraged and rely on recent HbA_1c_ levels, baseline survey scores, age, gender, race or ethnicity, diabetes duration, and diabetes device use as well as responses to the SMS text messaging surveys. Interviews are intended to further explore their approach to self-management using their diabetes devices. Interview prompts will focus on the participants’ daily approach to managing their diabetes, including decision-making, problem-solving, and interactions with technology. Alignment of interview prompts with the COM-B model is described in Table 1. Likewise, a portion of the interview will be dedicated to exploring study feasibility and acceptability, including perceived study burden and experiences of behavior change during the study period. Interviews will be conducted using Health Insurance Portability and Accountability Act (HIPAA)-compliant web conference tools (eg, Zoom; Zoom Communication, Inc), audio-video recorded, and transcribed for analysis. In line with a traditional qualitative approach, analysis and data collection will be completed simultaneously, and the final sample size will be determined by theoretical saturation.

### Analytic Approaches

### Overview

**Figure 3.**
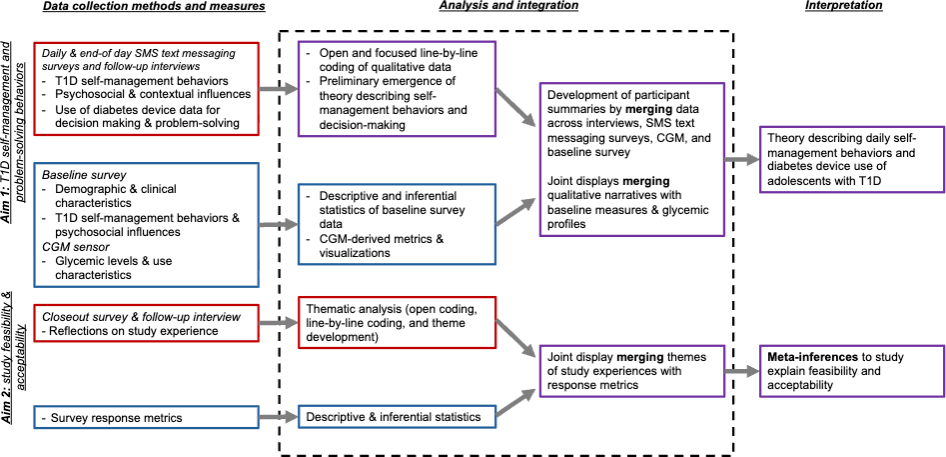
Overview of data analysis and integration plan. Red, blue, and purple colors indicate qualitative, quantitative, and mixed analyses, respectively. CGM: continuous glucose monitoring; T1D: type 1 diabetes.

Qualitative (open-ended survey responses and interview transcripts) and quantitative (baseline survey and CGM) data will be analyzed simultaneously and integrated to address the study aims. For aim 1, we will use a constructivist grounded theory approach, integrating findings from the daily survey and interview data with CGM and psychosocial measures. For aim 2, we will use a descriptive approach, integrating findings from the closeout survey and follow-up interview to assess feasibility and acceptability. Figure 3 summarizes the analysis and integration plan across data sources.

#### Qualitative Analysis

A .csv file with daily, end-of-day, and closeout survey responses will be downloaded from REDCap for each participant. Columns will include survey timing (eg, morning and afternoon) and study day followed by a column for each survey question. Each row will include responses from a unique survey instance. Interview transcripts and .csv files will be imported and grouped by participant within qualitative and mixed methods data analysis software to support data management (eg, MAXQDA [Max Weber Qualitative Data Analysis]; VERBI GmbH). Participant summaries will be created to merge all data by participant (see also “Mixed Methods Integration” section).

Following a constructivist grounded theory approach informed by Charmaz [42], the principal investigator and research team members will perform at least two iterative rounds of line-by-line coding on the qualitative data: (1) initial coding to identify concepts that are important to participants; and (2) focused coding to synthesize concepts and identify patterns across participants. Theoretical coding can be used to further examine relationships between codes to construct a theory grounded in participant experiences [42]. Throughout the grounded theory analysis, a hybrid inductive-deductive approach will be leveraged, incorporating domains from the COM-B model alongside those identified from participant responses [60] to assist in identifying critical self-management behaviors as they relate to diabetes device use. Findings will be presented as a theory of optimal advanced diabetes technology use for self-management, grounded in the experiences and perspectives of adolescents with T1D. We will refer to COREQ (Consolidated Criteria for Reporting Qualitative Research) guidelines Checklist 1to ensure rigor and transparency in reporting [61].

Due to the frequency of data collection in our approach, we anticipate that not all questions will be answered in the real-time daily surveys. We will look for patterns of missing data within and across participants to identify any systematic issues in data collection that may influence analysis and interpretation [62]. Otherwise, any missing data will be treated as a nonresponse. Due to the longitudinal nature of data collection, we anticipate having sufficient responses across daily survey and interview data to reach theoretical saturation. However, additional data collection will occur as needed to further refine the theory [42].

#### Quantitative Analysis

Baseline survey metrics and clinical characteristics collected from medical records (eg, HbA_1c_) and participant response rates will be summarized using descriptive statistics. In line with the adapted STROBE (Strengthening the Reporting of Observational Studies in Epidemiology) Checklist for Reporting EMA Studies (CREMAS) from Liao et al [63], we plan to report participant attrition rates according to study days, response latency, overall and daily compliance rates (total responses per participant and average number of responses), and associations of compliance with demographic, clinical, and other time-varying variables.

In this mixed methods study, quantitative measures will be used to characterize the sample and expand understanding of each participant’s experience when integrated with qualitative findings. CGM-derived metrics of glycemic control will include those recommended by consensus guidelines from Battelino et al [64] such as sensor use time, time in ranges (ie, above and below), and the glucose management indicator. A minimum of 14 days of CGM data with sensor use 70% of the time has been previously established as sufficient for analysis. The *iglu* R package (R Foundation for Statistical Computing) [65,66] will be used to process and analyze all collected CGM data, and interpolation will be used for small windows of missing data (eg, 45 min). Additional analysis packages (eg, *Best Linear IMPutation*) will be used to impute expected longer periods of missing data (ie, periods of signal loss and sensor malfunction) to reduce bias introduction and evaluate the randomness of missing data and impute missing values, where appropriate [67].

To test for mean differences in psychosocial and behavioral characteristics between glycemic level groups (HbA_1c_>9% and those with HbA_1c_≤9%) at baseline, 1-tailed *t* tests will be used. If the data are nonparametric, appropriate alternative tests (ie, the Wilcoxon rank paired test) will be performed. For all participants with at least 28 days of continuous glucose data, we will use linear mixed models to identify risk factors that influence blood glucose levels, model changes in blood glucose over time, and test any intervention effect resulting from longitudinal data collection. Additional statistical tests may be performed as appropriate based on the data collected. Statistical analysis will be performed on the data of all included study participants, and a statistical significance level of 5% (α=.05) will be used to test for differences and associations.

#### Mixed Methods Integration

Mixed methods findings will help us to better understand how diabetes devices are used for daily T1D self-management among adolescents with T1D and how psychosocial factors and daily self-management behaviors are related. Aligned with the longitudinal, convergent mixed methods design, integration of qualitative and quantitative approaches will occur through sampling, data collection, analysis, and interpretation [68,69]. For example, integration occurs during sampling by collecting data from identical samples in the qualitative and quantitative approaches. During data collection, participants will provide written reflections through SMS text messaging surveys (qualitative) on their glucose data from each day (quantitative). In addition, interview participants will provide narratives of their self-management behaviors that can be compared to CGM metrics.

During mixed methods analysis, we will first create participant summaries that include individual-level responses from interview transcripts, daily SMS text messaging surveys, baseline surveys, and CGM data. Raw and summary level baseline survey responses and summary level CGM metrics will be imported into the qualitative analysis software alongside the coded qualitative data. In addition, ambulatory glucose profiles from CGM data will be imported to provide context of recent and current glucose levels coinciding with real-time survey responses. We will then merge all data sources to create written participant summaries. Example participant profile provided in Multimedia Appendix 5.

We will also merge results to compare qualitative findings to diabetes outcomes (eg, self-management behaviors and diabetes distress) and glycemic levels. Using mixed methods features of MAXQDA software, we will create crosstabs to allow for comparison of qualitative findings by quantitative measures. For example, qualitative findings from interviews and daily SMS text messaging surveys will be compared between those with HbA_1c_ of more than 9% and those with HbA_1c_ of 9% or less. Integration through merging will allow us to identify patterns that can help to explain variation in glycemic levels or other diabetes-related physical and mental health outcomes. A similar approach will be used to integrate feasibility and acceptability data collected through the closeout survey and follow-up interview. Meta-inferences will be created to explain study feasibility and acceptability, including the perceived burden of participating in real-time and longitudinal data collection.

We will also create visual joint displays [69,70] that compare qualitative and quantitative results to generate meta-inferences. For example, one such joint display will examine patterns in qualitative findings by different glycemic outcomes to determine differences in the daily experiences of youth that help to explain glycemic levels and variability. A similar joint display can array qualitative findings by baseline measures of self-reported diabetes distress and self-management behaviors. Integrated findings will be used to further refine our theory of self-management with diabetes devices in this population.

When merging qualitative and quantitative results, we will determine whether the findings are concordant across approaches. Concordant results will be discussed among the research team, returning to the emergent grounded theory and comparing data and analytical categories (in qualitative findings) to quantitative results to examine meta-inferences. Both concordant and discordant results can support the refinement of the theory and generate additional hypotheses for future research [71-73].

## Results

This study was reviewed and approved by the institutional review board in January 2025 and enrolled its first participant in April 2025. As of December 2025, 25 participants have enrolled in this study, and we anticipate meeting our enrollment goal of approximately 30 to 40 participants in early 2026. We anticipate findings to become available in the following several years through conference presentations and peer-reviewed publications. Furthermore, findings will inform the development of an intervention to address diabetes self-management and decision-making in adolescents with T1D.

Few previous applications of real-time data collection have leveraged conversational SMS text messaging (where questions are sent one-at-a-time following participant responses), and, as such, there is little data on participant burden and compliance available for this method; however, early studies using closed-ended surveys indicate high satisfaction and compliance rates (≤95%) among adolescents and young adults [74], especially when fewer prompts were used [75,76]. As a result, our study incorporated several recommendations for burden reduction and compliance optimization such as working with a population that feels connected to our study objective, using shorter, less frequent prompts, allowing a window of 2+ hours for responses, allowing the use of participants’ own devices, providing a monetary incentive after completing the study, and co-developing surveys with youth with lived experience of T1D from the T1DYAC [77,78]. As a result, we are hopeful for response rates of at least 70% in this study. Likewise, we expect themes emerging from the analysis of feasibility and acceptability responses to highlight overall participant satisfaction and low burden.

We anticipate that sensor use time will be above 75%, as this population will already use CGM prior to study enrollment. In addition, we anticipate that participants with HbA_1c_ of more than 9% will report lower problem-solving, self-management behaviors, and diabetes device use, and higher diabetes distress and depression compared with those with HbA_1c_ of 9% or less.

Primary qualitative outcomes include thematic description of typical and momentary behaviors and responses to diabetes distress, problem-solving, and decision-making. Historically, reported adherence levels to real-time data collection (eg, quantitative ecological momentary assessment) among children and adolescents have been high (78%), including among youth using mobile devices and wearables (73%) [79]. We anticipate that, even with up to 30% missing data on daily surveys, the combined repeated surveys and in-depth interviews will provide a sufficient sample size to detect differences between adolescents with HbA_1c_ of 9% or less and more than 9%. However, monetary incentives are provided based upon the completion of the study and prorated based on the level of completion to incentivize data completeness. We anticipate that theoretical saturation will likely occur prior to the enrollment of 40 participants. When integrated, the quantitative and qualitative data sources will provide novel insights into daily self-management behaviors for adolescents with T1D.

## Discussion

Adolescents with T1D tend to struggle more with their diabetes self-management, have lower engagement with data provided by diabetes devices, and higher glycemic levels compared to all other age groups with this condition. Day-to-day self-management among adolescents may be further complicated by increasing independence from caregivers and ongoing physical, emotional, and cognitive development during this life stage. In particular, the ongoing development of executive functioning may inhibit optimal problem-solving abilities that are required for preventing and treating low and high blood glucose levels that arise throughout the day. Exploring routine, reactive, and reflective behaviors through real-time data collection can help us to better understand how adolescents make decisions and solve problems related to their diabetes during the day.

To our knowledge, this will be the first application of a real-time mixed methods approach to better understand daily decision-making and psychosocial influences surrounding diabetes self-management among adolescents with T1D. The robust data collection strategy has the potential to add significant value to the field as we work to better understand facilitators and barriers to optimal diabetes self-management in adolescents, especially among those who use CGM regularly. Furthermore, the qualitative insights provided will help elucidate intervention targets and preferences to improve self-management behaviors among this age group. Finally, this study will allow us to evaluate the feasibility of leveraging daily SMS text messaging for adolescents with T1D, which can open new potential avenues for both data collection and intervention.

While this study has several strengths, it does have limitations. First, this study protocol was limited by cohort geographics and direct recruitment from an academic medical center. However, based on the list generated from medical records and the flexible recruitment strategies, we intend to enroll a diverse sample of participants based on backgrounds, parental education level, and geographic location. While participants in this study did share structural challenges related to technology use (eg, access), future research should more intentionally explore the impact of these barriers on daily self-management experiences. Because this is one of the few applications of real-time qualitative data collection, there are still methodological questions to address, including potential participant burden, impact of data collection on participant behavior, and limitations to data quality. Findings related to feasibility and acceptability will be used to address these methodological questions and improve future studies using qualitative data collection through SMS text messaging and other real-time techniques. Recruitment and data collection for this study began in Spring 2025 and will likely continue through early 2026.

This study will be among the first to use a mixed methods, real-time approach and leverage SMS text messaging among adolescents with T1D. We anticipate that this approach will bring forth new insights into the daily self-management and problem-solving behaviors of adolescents with T1D. In addition, the combination of several data sources including glucose levels and semistructured interviews will allow for comparison of glycemic outcomes with the self-reported lived experiences of adolescents with T1D. This integrated approach will give a new perspective on self-management by adding contextual information to objective glucose data, supporting identification of key barriers to self-management in this population. Findings will inform future studies and interventions aiming to address diabetes self-management behaviors and problem-solving among adolescents with T1D.

## Supplementary material

10.2196/83218 Multimedia Appendix 1 Demographics and clinical characteristics survey from the Qualitative Insights to Type 1 Diabetes in Youth (QUALITY) Study.

10.2196/83218 Multimedia Appendix 2 Validated instruments included in Qualitative Insights to Type 1 Diabetes in Youth (QUALITY) baseline survey.

10.2196/83218 Multimedia Appendix 3 Real-time SMS text messaging survey questions.

10.2196/83218 Multimedia Appendix 4 Qualitative Insights to Type 1 Diabetes in Youth (QUALITY) closeout survey.

10.2196/83218 Multimedia Appendix 5 Sample participant profile.

10.2196/83218 Checklist 1 COREQ checklist.

## Abbreviations

CGM: continuous glucose monitoring
COREQ: Consolidated Criteria for Reporting Qualitative Research
CREMAS: Checklist for Reporting EMA Studies
EMERSE: Electronic Medical Record Search Engine
HbA_1c_: hemoglobin A_1c_
HIPAA: Health Insurance Portability and Accountability Act
MAXQDA: Max Weber Qualitative Data Analysis
QUALITY: Qualitative Insights to Type 1 Diabetes in Youth
REDCap: Research Electronic Data Capture
STROBE: Strengthening the Reporting of Observational Studies in Epidemiology
T1D: type 1 diabetes
T1DYAC: Type 1 Diabetes Youth Advisory Council
U-M: University of Michigan

## References

[1] Fang M, Wang D, Selvin E (2024). Prevalence of type 1 diabetes among US children and adults by age, sex, race, and ethnicity. JAMA.

[2] Gong B, Yang W, Xing Y, Lai Y, Shan Z (2025). Global, regional, and national burden of type 1 diabetes in adolescents and young adults. Pediatr Res.

[3] American Diabetes Association Professional Practice Committee (2025). 14. Children and adolescents: standards of care in diabetes-2025. Diabetes Care.

[4] Redondo MJ, Libman I, Maahs DM (2021). The evolution of hemoglobin A_1c_ targets for youth with type 1 diabetes: rationale and supporting evidence. Diabetes Care.

[5] Fang M, Xu Y, Ballew SH (2025). Trends and disparities in technology use and glycemic control in type 1 diabetes. JAMA Netw Open.

[6] Miller KM, Beck RW, Foster NC, Maahs DM, for the T1D Exchange (2020). HbA1c levels in type 1 diabetes from early childhood to older adults: a deeper dive into the influence of technology and socioeconomic status on HbA1c in the T1D exchange clinic registry findings. Diabetes Technol Ther.

[7] Anderzén J, Samuelsson U, Gudbjörnsdottir S, Hanberger L, Åkesson K (2016). Teenagers with poor metabolic control already have a higher risk of microvascular complications as young adults. J Diabetes Complicat.

[8] Azar S, Maroun Abou Jaoude N, Kędzia A, Niechciał E (2024). Barriers to type 1 diabetes adherence in adolescents. J Clin Med.

[9] Hofer SE, Raile K, Fröhlich-Reiterer E (2014). Tracking of metabolic control from childhood to young adulthood in type 1 diabetes. J Pediatr.

[10] Hill-Briggs F, Adler NE, Berkowitz SA (2020). Social determinants of health and diabetes: a scientific review. Diabetes Care.

[11] Young-Hyman D, de Groot M, Hill-Briggs F, Gonzalez JS, Hood K, Peyrot M (2016). Psychosocial care for people with diabetes: a position statement of the American Diabetes Association. Diabetes Care.

[12] Zuijdwijk CS, Cuerden M, Mahmud FH (2013). Social determinants of health on glycemic control in pediatric type 1 diabetes. J Pediatr.

[13] Annan SF, Higgins LA, Jelleryd E (2022). ISPAD Clinical Practice Consensus Guidelines 2022: Nutritional management in children and adolescents with diabetes. Pediatr Diabetes.

[14] Ding Y, Zhang W, Wu X (2022). Deterioration in glycemic control on schooldays among children and adolescents with type 1 diabetes: a continuous glucose monitoring-based study. Front Pediatr.

[15] Baucom KJW, Queen TL, Wiebe DJ (2015). Depressive symptoms, daily stress, and adherence in late adolescents with type 1 diabetes. Health Psychol.

[16] Chiang JL, Maahs DM, Garvey KC (2018). Type 1 diabetes in children and adolescents: a position statement by the American Diabetes Association. Diabetes Care.

[17] Hagger V, Hendrieckx C, Sturt J, Skinner TC, Speight J (2016). Diabetes distress among adolescents with type 1 diabetes: a systematic review. Curr Diab Rep.

[18] Luo J, Wang H, Li X (2021). Factors associated with diabetes distress among adolescents with type 1 diabetes. J Clin Nurs.

[19] Hagger V, Hendrieckx C, Cameron F, Pouwer F, Skinner TC, Speight J (2018). Diabetes distress is more strongly associated with HbA1c than depressive symptoms in adolescents with type 1 diabetes: results from diabetes MILES youth-Australia. Pediatr Diabetes.

[20] Spitzer RL, Kroenke K, Williams JB (1999). Validation and utility of a self-report version of PRIME-MD: the PHQ primary care study. Primary Care Evaluation of Mental Disorders. Patient Health Questionnaire. JAMA.

[21] Ziegler R, von Sengbusch S, Kröger J (2019). Therapy adjustments based on trend arrows using continuous glucose monitoring systems. J Diabetes Sci Technol.

[22] DeSalvo DJ, Miller KM, Hermann JM (2018). Continuous glucose monitoring and glycemic control among youth with type 1 diabetes: international comparison from the T1D exchange and DPV initiative. Pediatr Diabetes.

[23] Huhn F, Lange K, Jördening M, Ernst G (2023). Real-world use of continuous glucose monitoring systems among adolescents and young adults with type 1 diabetes: reduced burden, but little interest in data analysis. J Diabetes Sci Technol.

[24] Brew-Sam N, Chhabra M, Parkinson A (2021). Experiences of young people and their caregivers of using technology to manage type 1 diabetes mellitus: systematic literature review and narrative synthesis. JMIR Diabetes.

[25] Tanenbaum ML, Commissariat PV (2023). Experience with burdens of diabetes device use that affect uptake and optimal use in people with type 1 diabetes. Endocr Connect.

[26] Dowd R, Jepson LH, Green CR, Norman GJ, Thomas R, Leone K (2023). Glycemic outcomes and feature set engagement among real-time continuous glucose monitoring users with type 1 or non-insulin-treated type 2 diabetes: retrospective analysis of real-world data. JMIR Diabetes.

[27] Laffel LM, Kanapka LG, Beck RW (2020). Effect of continuous glucose monitoring on glycemic control in adolescents and young adults with type 1 diabetes: a randomized clinical trial. JAMA.

[28] Sheikh K, Bartz SK, Lyons SK, DeSalvo DJ (2018). Diabetes device use and glycemic control among youth with type 1 diabetes: a single-center, cross-sectional study. J Diabetes Res.

[29] Divilly P, Zaremba N, Mahmoudi Z (2022). Hypo-METRICS: Hypoglycaemia-MEasurement, ThResholds and ImpaCtS—A multi-country clinical study to define the optimal threshold and duration of sensor-detected hypoglycaemia that impact the experience of hypoglycaemia, quality of life and health economic outcomes: the study protocol. Diabet Med.

[30] Mulvaney SA, Vaala SE, Carroll RB (2019). A mobile app identifies momentary psychosocial and contextual factors related to mealtime self-management in adolescents with type 1 diabetes. J Am Med Inform Assoc.

[31] Nam S, Griggs S, Ash GI (2021). Ecological momentary assessment for health behaviors and contextual factors in persons with diabetes: a systematic review. Diabetes Res Clin Pract.

[32] Zhang P, Fonnesbeck C, Schmidt DC, White J, Kleinberg S, Mulvaney SA (2022). Using momentary assessment and machine learning to identify barriers to self-management in type 1 diabetes: observational study. JMIR Mhealth Uhealth.

[33] Ehrmann D, Schmitt A, Priesterroth L, Kulzer B, Haak T, Hermanns N (2022). Time with diabetes distress and glycemia-specific distress: new patient-reported outcome measures for the psychosocial burden of diabetes using ecological momentary assessment in an observational study. Diabetes Care.

[34] Michie S, van Stralen MM, West R (2011). The behaviour change wheel: a new method for characterising and designing behaviour change interventions. Implement Sci.

[35] Butler AM, Hilliard ME, Titus C (2020). Barriers and facilitators to involvement in children’s diabetes management among minority parents. J Pediatr Psychol.

[36] Cockcroft EJ, Wooding EL, Narendran P (2023). Factors affecting the support for physical activity in children and adolescents with type 1 diabetes mellitus: a national survey of health care professionals’ perceptions. BMC Pediatr.

[37] McSharry J, Byrne M, Casey B (2020). Behaviour change in diabetes: behavioural science advancements to support the use of theory. Diabet Med.

[38] Hamilton K, Stanton-Fay SH, Chadwick PM (2021). Sustained type 1 diabetes self-management: specifying the behaviours involved and their influences. Diabet Med.

[39] Smith R, Eisenberg S, Turner-Pfifer A (2024). We are on the verge of breakthrough cures for type 1 diabetes, but who are the 2 million Americans who have it?. J Health Econ Outcomes Res.

[40] Hanauer DA, Barnholtz-Sloan JS, Beno MF (2020). Electronic Medical Record Search Engine (EMERSE): an information retrieval tool for supporting cancer research. JCO Clin Cancer Inform.

[41] Hanauer DA, Mei Q, Law J, Khanna R, Zheng K (2015). Supporting information retrieval from electronic health records: a report of University of Michigan’s nine-year experience in developing and using the electronic medical record search engine (EMERSE). J Biomed Inform.

[42] Charmaz K (2006). Constructing Grounded Theory.

[43] Hennink MM, Kaiser BN, Marconi VC (2017). Code saturation versus meaning saturation: how many interviews are enough?. Qual Health Res.

[44] Malterud K, Siersma VD, Guassora AD (2016). Sample size in qualitative interview studies: guided by information power. Qual Health Res.

[45] Saunders B, Sim J, Kingstone T (2018). Saturation in qualitative research: exploring its conceptualization and operationalization. Qual Quant.

[46] Onwuegbuzie AJ, Johnson RB, Collins KMT (2011). Assessing legitimation in mixed research: a new framework. Qual Quant.

[47] Perez A, Howell Smith MC, Babchuk WA, Lynch-O’Brien LI (2023). Advancing quality standards in mixed methods research: extending the legitimation typology. J Mix Methods Res.

[48] Harris PA, Taylor R, Minor BL (2019). The REDCap consortium: building an international community of software platform partners. J Biomed Inform.

[49] Harris PA, Taylor R, Thielke R, Payne J, Gonzalez N, Conde JG (2009). Research Electronic Data Capture (REDCap)—a metadata-driven methodology and workflow process for providing translational research informatics support. J Biomed Inform.

[50] Iannotti RJ, Schneider S, Nansel TR (2006). Self-efficacy, outcome expectations, and diabetes self-management in adolescents with type 1 diabetes. J Dev Behav Pediatr.

[51] Rasbach LE, Volkening LK, Markowitz JT, Butler DA, Katz ML, Laffel LMB (2015). Youth and parent measures of self-efficacy for continuous glucose monitoring: survey psychometric properties. Diabetes Technol Ther.

[52] Tanenbaum ML, Hanes SJ, Miller KM, Naranjo D, Bensen R, Hood KK (2017). Diabetes device use in adults with type 1 diabetes: barriers to uptake and potential intervention targets. Diabetes Care.

[53] Messer LH, Cook PF, Tanenbaum ML, Hanes S, Driscoll KA, Hood KK (2019). CGM benefits and burdens: two brief measures of continuous glucose monitoring. J Diabetes Sci Technol.

[54] Weissberg-Benchell J, Antisdel-Lomaglio J (2011). Diabetes-specific emotional distress among adolescents: feasibility, reliability, and validity of the problem areas in diabetes-teen version. Pediatr Diabetes.

[55] Richardson LP, Rockhill C, Russo JE (2010). Evaluation of the PHQ-2 as a brief screen for detecting major depression among adolescents. Pediatrics.

[56] Mehta SN, Nansel TR, Volkening LK, Butler DA, Haynie DL, Laffel LMB (2015). Validation of a contemporary adherence measure for children with type 1 diabetes: the diabetes management questionnaire. Diabet Med.

[57] Mulvaney SA, Jaser SS, Rothman RL (2014). Development and validation of the diabetes adolescent problem solving questionnaire. Patient Educ Couns.

[58] Sidoti O, Park E, Faverio M, Atske S, Radde K Teens and internet, device access fact sheet. Pew Research Center.

[59] Sun X, Haydel KF, Matheson D, Desai M, Robinson TN (2023). Are mobile phone ownership and age of acquisition associated with child adjustment? A 5-year prospective study among low-income Latinx children. Child Dev.

[60] Proudfoot K (2023). Inductive/deductive hybrid thematic analysis in mixed methods research. J Mix Methods Res.

[61] Tong A, Sainsbury P, Craig J (2007). Consolidated Criteria for Reporting Qualitative Research (COREQ): A 32-item checklist for interviews and focus groups. Int J Qual Health Care.

[62] Morse JM (2011). The case of the missing data. Qual Health Res.

[63] Liao Y, Skelton K, Dunton G, Bruening M (2016). A systematic review of methods and procedures used in ecological momentary assessments of diet and physical activity research in youth: an adapted STROBE Checklist for Reporting EMA Studies (CREMAS). J Med Internet Res.

[64] Battelino T, Alexander CM, Amiel SA (2023). Continuous glucose monitoring and metrics for clinical trials: an international consensus statement. Lancet Diabetes Endocrinol.

[65] Broll S, Urbanek J, Buchanan D (2021). Interpreting blood GLUcose data with R package iglu. PLoS ONE.

[66] Chun E, Fernandes NJ, Gaynanova I (2024). An update on the iglu software package for interpreting continuous glucose monitoring data. Diabetes Technol Ther.

[67] Olsen MT, Klarskov CK, Dungu AM, Hansen KB, Pedersen-Bjergaard U, Kristensen PL (2025). Statistical packages and algorithms for the analysis of continuous glucose monitoring data: a systematic review. J Diabetes Sci Technol.

[68] Fetters MD, Molina-Azorin JF (2017). The journal of mixed Methods research starts a new decade: the mixed methods research integration trilogy and its dimensions. J Mix Methods Res.

[69] Guetterman TC, Manojlovich M (2024). Grand rounds in methodology: designing for integration in mixed methods research. BMJ Qual Saf.

[70] Guetterman TC, Fetters MD, Creswell JW (2015). Integrating quantitative and qualitative results in health science mixed methods research through joint displays. Ann Fam Med.

[71] Bazeley P (2018). Integrating Analyses in Mixed Methods Research.

[72] Fetters MD (2020). The Mixed Methods Research Workbook: Activities for Designing, Implementing, and Publishing Projects.

[73] Younas A, Pedersen M, Inayat S (2023). Practical strategies to identify and address discordant findings in mixed methods research. Res Methods Med Health Sci.

[74] Psihogios AM, Li Y, Ahmed A (2021). Daily text message assessments of 6‐mercaptopurine adherence and its proximal contexts in adolescents and young adults with leukemia: a pilot study. Pediatr Blood Cancer.

[75] Drexl K, Ralisa V, Rosselet-Amoussou J (2025). Readdressing the ongoing challenge of missing data in youth ecological momentary assessment studies: meta-analysis update. J Med Internet Res.

[76] Wang S, Yang CH, Brown D, Cheng A, Kwan MYW (2025). Participant compliance with ecological momentary assessment in movement behavior research among adolescents and emerging adults: systematic review. JMIR Mhealth Uhealth.

[77] Murray AL, Xie T, Power L, Condon L (2024). Recruitment and retention of adolescents for an ecological momentary assessment measurement burst mental health study: the MHIM engagement strategy. Health Expect.

[78] Smyth JM, Jones DR, Wen CKF, Materia FT, Schneider S, Stone A (2021). Influence of ecological momentary assessment study design features on reported willingness to participate and perceptions of potential research studies: an experimental study. BMJ Open.

[79] Wen CKF, Schneider S, Stone AA, Spruijt-Metz D (2017). Compliance with mobile ecological momentary assessment protocols in children and adolescents: a systematic review and meta-analysis. J Med Internet Res.

