# Supplementary material for: Real-Time Type 1 Diabetes Self-Management Decision-Making in Adolescents: Protocol for a Longitudinal Mixed Methods Study Using Text Messaging and Continuous Glucose Monitoring
Source: JMIR Res Protoc. 2026 Mar 4;15:e83218. doi: 10.2196/83218 (PMC12978980; doi:10.2196/83218)
Supplement: Multimedia Appendix 1 [file resprot-v15-e83218-s001.docx]

**Multimedia Appendix 1.** Demographics and clinical characteristics survey from QUALITY Study

General Demographics

1. How would you describe your gender?
2. Which of the following best describe your gender (Select all that apply):
   - Girl/woman/feminine
   - Boy/man/masculine
   - Nonbinary
   - Agender/no gender
   - Questioning
   - I don’t know what this question is asking
   - I prefer not to answer this question
3. Are you transgender?
   - Yes
   - No
   - Questioning
   - I don’t know what this question is asking
   - I prefer not to answer this question
4. How do you describe your race, ethnicity, tribal affiliation, country of origin, or ancestry? [Open]
5. Which of the following best describes your background (also known as race/ethnicity)? Select all that apply.
   - Hispanic/Latino/a/x
     - Central American
     - Mexican
     - South American
     - I identify as Hispanic or Latino/a/x, but not as one of the groups listed above
   - Asian
     - Asian Indian
     - Cambodian
     - Chinese
     - Communities of Myanmar
     - Filipino/a
     - Hmong
     - Japanese
     - Korean
     - Laotian
     - South Asian
     - Vietnamese
     - I identify as Asian, but not as one of the groups listed above
   - Middle Eastern or North African
     - Middle Eastern
     - Arabic
     - North African
     - I identify as Middle Eastern or North African, but not as one of the groups listed above
   - Black
     - African American
     - Afro-Caribbean
     - Ethiopian
     - Somali
     - I identify as African, but not as one of the groups listed above
     - I identify as Black, but not as one of the groups listed above
   - American Indian/Alaska Native
     - American Indian
     - Alaska Native
     - Canadian Inuit, Metis, or First Nation
     - I identify as Indigenous Mexican, Central American, or South American, but not as one of the groups listed above
   - White
     - Eastern European
     - Slavic
     - Western European
     - I identify as White, but not as one of the groups listed above
   - Native Hawaiian or Pacific Islander
     - Chamoru (Chamorro)
     - Marshallese
     - Communities of the Micronesian Region
     - Native Hawaiian
     - Samoan
     - I identify as Native Hawaiian or Pacific Islander, but not as one of the groups listed above
6. We realize we may have not captured everything about you and your identity. If you would like to say more about communities you represent, please feel free to share.
7. How would you describe your disability status? We are interested regardless of whether you typically request accommodations for this disability. Please select all that apply.
   - Deaf or serious difficulty hearing
   - Blind or serious difficulty seeing (even when wearing glasses)
   - Serious difficulty walking or climbing stairs
   - Difficulty concentrating, remembering, or making decisions
   - Difficulty dressing or bathing
   - Using your usual (customary) language, have serious difficulty communicating or being understood
   - I am not comfortable disclosing/I do not wish to disclose my disability status.
   - I do not identify with a disability or impairment
   - A disability or impairment not listed above (please describe)
8. Are you currently a student?
   - Yes
   - No
9. (If yes to previous) Which of the following best describes your current student status?
   - Middle school student
   - High school student
   - Part-time college student
   - Full-time college student
   - Trade or vocational school training
   - Other
10. Which of the following best describes your current employment status?
    - Not currently working for pay
    - Unable to work due to disability
    - Working part time (<20 hours per week)
    - Working part time (20-35 hours per week)
    - Working full time (35+ hours per week)

Diabetes History

1. How many years ago were you diagnosed by a doctor with diabetes?
   - 0-18
2. In the last twelve months, did you ever need emergency medical attention for your diabetes? This might include going to the emergency room, calling an ambulance, spending a night in the hospital, etc.
   - Yes
   - No
   - (If yes, why did you go to the hospital?)
     - Diabetic Ketoacidosis (DKA) or high blood sugar
     - Hypoglycemia or low blood sugar
3. Which of the following medical professionals helps you to manage your diabetes? (Select all that apply).
   - Adult endocrinologist
   - Pediatric endocrinologist
   - Primary care provider
   - A different provider (please specify)
   - I do not currently have a provider who helps me manage my diabetes
4. (If peds endo) Do you anticipate transitioning to adult endocrinology in the next year?
   - Yes
   - No
5. How often in the last year have you seen a doctor or other provider for your diabetes?
   - 0 times
   - 1 time
   - 2 times
   - 3 times
   - 4 or more times
6. How often in the last year have you seen a diabetes educator for your diabetes?
   - 0 times
   - 1 time
   - 2 times
   - 3 times
   - 4 or more times
7. Does someone typically attend your diabetes-related appointments with you?
   - No, I go to my appointments alone
   - Yes, a parent goes with me
   - Yes, a spouse or significant other attends with me
   - Yes, a friend or support person attends with me
   - Other (please specify)
8. Who helps you with your day-to-day diabetes management? (Select all that apply)
   - Parent
   - Other family member(s)
   - Friends
   - Significant other (boyfriend/girlfriend, spouse)
   - Therapist, counselor, or social worker
   - Diabetes medical team (nurse, doctor, etc.)
   - Online resources (website, blog, etc.)
   - Social Media or other online community
   - Nobody
9. Who helps you manage the emotional challenges of having diabetes? (Select all that apply)
   - Parent
   - Other family member(s)
   - Friends
   - Significant other
   - Therapist, counselor, or social worker
   - Diabetes medical team (nurse, doctor, etc.)
   - Online resources (website, blog, etc.)
   - Social Media or other online community
   - Nobody

Diabetes Technology

1. What type of continuous glucose monitor (or “CGM”) do you currently use?
   - Freestyle Libre 14-day (oldest edition)
   - Freestyle Libre 2
   - Freestyle Libre 3
   - Dexcom G6
   - Dexcom G7
   - Medtronic Guardian 3
   - Medtronic Guardian connect
   - Other
2. For how long have you used a CGM?
   - 0-3 months
   - 4-6 months
   - 7-12 months
   - 1-3 years
   - 4-6 years
   - More than 6 years
3. How many days in the last month did you wear/use your CGM in the last month?
   - fewer than 8 days of the month
   - between 8 days and 16 days
   - Between 16 days and 24 days
   - More than 24 days of the month
4. Are you currently using an insulin pump? (Yes, No)
   - What kind of insulin pump/closed loop system are you currently using?
     - Medtronic MiniMed
     - Medtronic 670 or 770
       - How often is your pump set to “auto mode”?
         - Never
         - Less than 25% of the time (fewer than 6 hours per day)
         - Between 25% and 50% of the time (about 6-12 hours per day)
         - Between 50% and 75% of the time (about 12-18 hours per day)
         - More than 75% of the time (more than 18 hours per day)
     - Medtronic 630
     - Older Medtronic Model (e.g., Paradigm Revel 723)
     - Tandem t:slim
       - → Do you use Control IQ?
         - Yes
         - No
       - → If yes, how often are you using the Control IQ mode?
         - never
         - less than 25% of the time
         - 25-50% of the time
         - 50-75% of the time
         - >75% of the time
       - → If no, do you use Basal IQ?
         - Yes
         - No
       - Omnipod Eros
       - Omnipod Dash
       - Omnipod 5 (OP5)
         - → How often are you in “auto mode”?

never

less than 25%

25-50%

50-75%

>75%

- - Do you regularly use any of the following methods to administer insulin?
    - Insulin vials and syringes
    - Oral insulin
    - Traditional insulin pens
    - Smart insulin pen (with a Bluetooth connection)
    - Inhalable insulin (e.g., Afrezza)
    - Needle-free (“jet”) insulin injectors
    - Something else (please describe)

1. Are there any other tools you use to help you manage your diabetes? (e.g., a tracking app on your phone, a glucometer, etc.)? Tell us about it.
2. Do you have any other health conditions that might impact your diabetes and/or your ability to care for your diabetes? Tell us about it.
