# Supplementary material for: Real-Time Type 1 Diabetes Self-Management Decision-Making in Adolescents: Protocol for a Longitudinal Mixed Methods Study Using Text Messaging and Continuous Glucose Monitoring
Source: JMIR Res Protoc. 2026 Mar 4;15:e83218. doi: 10.2196/83218 (PMC12978980; doi:10.2196/83218)
Supplement: Multimedia Appendix 2 [file resprot-v15-e83218-s002.docx]

**Multimedia Appendix 2.** Validated Instruments Included in QUALITY Baseline Survey

*Adherence to Diabetes Self-Management Behaviors*

Daily self-management of diabetes requires both the regular performance of several behaviors including blood glucose checking, insulin dosing, and treatment or correction of low and high blood glucose levels. While perfect adherence to self-management behaviors is not realistic, higher adherence is predictive of better glycemic outcomes in all ages with T1D. The Diabetes Management Questionnaire (DMQ) is a contemporary measure of adherence to diabetes management behavior developed by a multidisciplinary team of pediatric endocrinologists, nurses, educators, registered dieticians, and behavioral and nutrition scientists for use in pediatric T1D populations [1]. The 20-item scale includes subscales for behaviors associated with physical activity, meal and snack times, low and high blood glucose, and insulin use and checking blood glucose in the past month and is applicable to CGM users. Respondents self-report adherence on a defined 5-point scale (1 = almost never, 5 = almost always).

*Self-Efficacy for T1D Self-Management*

Within the Social Cognitive Theory, Bandura describes both self-efficacy and outcome expectations as key factors in one’s likelihood of completing behaviors. While self-efficacy refers to one’s belief that they are capable, outcome expectations describe short- and long-term benefits and consequences of performing behaviors. In the context of T1D, adolescents who are confident in managing their diabetes have lower glycemic levels [2, 3]. Likewise, it would also make sense that adolescents who believe there are benefits to engaging in diabetes self-management behaviors will be more motivated to perform them [3]. The Self-Efficacy for Diabetes Management- Short Form developed and validated by Iannotti and colleagues (2006) to evaluate self-efficacy and outcome expectations among youth with T1D [3]. Items within the self-efficacy subscale are rated from 0 (not sure at all) to 10 (completely sure), while those within the outcome expectations subscales are rated from 0 (not at all) to 10 (a lot). Due to the importance of technology use in this study, in addition to regular diabetes self-management behaviors, self-efficacy with diabetes devices will also be assessed using a modified version of the CGM Self-Efficacy scale [4]. Each of the 10 selected items will be rated on a 7-point Likert scale (0 = strongly disagree, 6 = strongly agree).

*Attitudes Towards Diabetes Technology*

Satisfaction with and attitudes towards diabetes technology are important predictors of device adherence among individuals with T1D [5]. The Benefits of CGM (BenCGM) and Burdens of CGM (BurCGM) scales were developed and validated by Messer and colleagues to evaluate the perceived benefits and burdens of CGM use among adolescents with T1D [5]. Items from both scales represent real-life benefits and challenges of CGM use such as cost, trust, alerts, and understanding the data provided. Each of the 8-item scales is rated by participants on a 5-point Likert scale from 1 (strongly disagree) to 5 (strongly agree). In addition, the Diabetes Technology Attitudes Scale (DTAS), developed by Tanenbaum and colleagues (2017) to examine reasons for and factors associated with diabetes device uptake, use, and discontinuation [6]. The brief 5-item scale focuses on perceptions of how diabetes technology improves or burdens the respondent’s life. Responses are self-reported using a 5-point Likert scale (1 = strongly disagree, 5 = strongly agree).

*Diabetes Self-Management Problem Solving*

In addition to the recommended self-management behaviors, individuals must also be prepared to prevent and solve problems that arise throughout the day such as preventing low and high blood sugars through diet, exercise, and insulin use [7]. The 13-item Diabetes Adolescent Problem-Solving Questionnaire (DAPSQ) was developed as a brief self-report measure of diabetes self-management problem solving for adolescents with T1D for clinical and research use. Items focus on responses and prevention of common diabetes problems including low and high blood sugars and maintenance in various settings and are rated on a defined 6-point scale (0 = never, 5 = always).

*Mood*

Among adolescents with T1D, depression contributes to lower adherence to self-management and higher glycemic levels [8]. The 9-item Patient Health Questionnaire (PHQ-9) is commonly used in clinical settings with adults and adolescents to screen for depression [9]. In this study, the two-item version (PHQ-2) designed for use in adolescents will be used to characterize depressive symptoms among our sample [10]. In the PHQ-2, participants are asked to rate how often they were bothered during the past two weeks by 1) feeling down, depressed, or hopeless, and 2) having little interest or pleasure in doing things on a 4-point scale from 0 (not at all) to 3 (nearly every day).

Higher levels of diabetes distress have been associated with higher glycemic levels among both adolescents with T1D [11] T1D. The Problem Areas in Diabetes- Teen Version (PAID-T) was developed and validated among 11-to-19-year-olds by Weissberg-Benchell and colleagues to measure the lived experiences of anxiety, overwhelm, and burden associated with the daily self-management of diabetes in adolescents [12]. The PAID-T scale is commonly used in pediatric diabetes clinics with children and adolescents with T1D. The 26-item PAID-T will be used to evaluate diabetes distress among this sample. Items focus on common diabetes management challenges and worries including being overwhelmed by the diabetes regimen, the frequency of checking blood glucose, weight, diet, high and lows, and motivation. Each item on the scale is rated by the participant on a 6-point Likert scale from 1 (not a problem) to 6 (serious problem).

**Table 1.** Summary of variables of interest and instruments from QUALITY baseline survey

| Variable of interest | Instrument | Sample items & scale |
| --- | --- | --- |
| Adherence to diabetes self-management behaviors | Diabetes Management Questionnaire (DMQ) [1] | How often did you or your parent / guardian…  Use the amount of carbohydrate to help decide the amount of insulin to give for a meal?  1 = almost never, 5 = almost always |
| Self-Efficacy for T1D Self-Management | Self-Efficacy for Diabetes Management-Short Form (SEDM-SF) [3] | Self-efficacy subscale: how sure are you that you can do each of the following, almost all the time?  Adjust your insulin or food accurately based on how much exercise you get.  0 = not sure at all, 10 = completely sure  Outcome expectations subscale: ‘If I do everything I’m supposed to do to take care of my diabetes, it would...”  1) Make me have fewer high blood sugars.  2) Be too much responsibility.  0 = not at all, 10 = a lot |
|  | CGM Self-Efficacy Scale [4] | I am sure I can...  1) Look at my current blood glucose levels in the mobile app/ receiver  0 = strongly disagree, 6 = strongly agree |
| Attitudes towards diabetes technologies | Benefits of CGM (BenCGM) and Burdens of CGM (BurCGM) [5] | 1) CGM makes taking care of diabetes easier  2) CGM sensor readings cannot be trusted  1 = strongly disagree, 5 = strongly agree |
|  | Diabetes Technology Attitudes Scale (DTAS) [6] | 1) Diabetes technology has made my life better  2) Diabetes technology has made my life easier  1 = strongly disagree, 5 = strongly agree |
| Diabetes self-management problem solving behaviors | Diabetes Adolescent Problem-Solving Questionnaire (DAPSQ) [7] | 1) I try to predict what will happen if I choose a certain solution to a diabetes problem.  2) If a solution to a diabetes problem doesn’t work, I try to figure out why.  0 = never, 5 = always |
| Mood | Patient Health Questionnaire- Adolescent 2-Item (PHQ-2) Version [10] | How often were you bothered during the past two weeks by:  1) feeling down, depressed, or hopeless?  2) having little interest or pleasure in doing things?  0 = not at all, 3 = nearly every day |
|  | Problem Areas in Diabetes – Teen Version (PAID-T) [12] | 1) Feeling overwhelmed by my diabetes regimen.  2) Feeling angry when I think about having and living with diabetes.  1 = not a problem, 6 = serious problem |

**Multimedia Appendix 2 References**

1. Mehta SN, Nansel TR, Volkening LK, Butler DA, Haynie DL, Laffel LM. Validation of a contemporary adherence measure for children with Type 1 diabetes: the Diabetes Management Questionnaire. Diabet Med. 2015 Sep;32(9):1232-8. PMID: 26280463. doi: 10.1111/dme.12682.

2. Guo J, Yang J, Wiley J, Ou X, Zhou Z, Whittemore R. Perceived stress and self-efficacy are associated with diabetes self-management among adolescents with type 1 diabetes: A moderated mediation analysis. J Adv Nurs. 2019 Dec;75(12):3544-53. PMID: 31441523. doi: 10.1111/jan.14179.

3. Iannotti RJ, Schneider S, Nansel TR, Haynie DL, Plotnick LP, Clark LM, et al. Self-efficacy, outcome expectations, and diabetes self-management in adolescents with type 1 diabetes. J Dev Behav Pediatr. 2006 Apr;27(2):98-105. PMID: 16682872. doi: 10.1097/00004703-200604000-00003.

4. Rasbach LE, Volkening LK, Markowitz JT, Butler DA, Katz ML, Laffel LM. Youth and parent measures of self-efficacy for continuous glucose monitoring: survey psychometric properties. Diabetes Technol Ther. 2015 May;17(5):327-34. PMID: 25695341. doi: 10.1089/dia.2014.0366.

5. Messer LH, Cook PF, Tanenbaum ML, Hanes S, Driscoll KA, Hood KK. CGM Benefits and Burdens: Two Brief Measures of Continuous Glucose Monitoring. J Diabetes Sci Technol. 2019 Nov;13(6):1135-41. PMID: 30854886. doi: 10.1177/1932296819832909.

6. Tanenbaum ML, Hanes SJ, Miller KM, Naranjo D, Bensen R, Hood KK. Diabetes Device Use in Adults With Type 1 Diabetes: Barriers to Uptake and Potential Intervention Targets. Diabetes Care. 2017 Feb;40(2):181-7. PMID: 27899489. doi: 10.2337/dc16-1536.

7. Mulvaney SA, Jaser SS, Rothman RL, Russell WE, Pittel EJ, Lybarger C, et al. Development and validation of the diabetes adolescent problem solving questionnaire. Patient Educ Couns. 2014 Oct;97(1):96-100. PMID: 25063715. doi: 10.1016/j.pec.2014.07.005.

8. Hood KK, Huestis S, Maher A, Butler D, Volkening L, Laffel LM. Depressive symptoms in children and adolescents with type 1 diabetes: association with diabetes-specific characteristics. Diabetes Care. 2006 Jun;29(6):1389-91. PMID: 16732028. doi: 10.2337/dc06-0087.

9. Spitzer RL, Kroenke K, Williams JB. Validation and utility of a self-report version of PRIME-MD: the PHQ primary care study. Primary Care Evaluation of Mental Disorders. Patient Health Questionnaire. JAMA. 1999 Nov 10;282(18):1737-44. PMID: 10568646. doi: 10.1001/jama.282.18.1737.

10. Richardson LP, Rockhill C, Russo JE, Grossman DC, Richards J, McCarty C, et al. Evaluation of the PHQ-2 as a brief screen for detecting major depression among adolescents. Pediatrics. 2010 May;125(5):e1097-103. PMID: 20368315. doi: 10.1542/peds.2009-2712.

11. Hagger V, Hendrieckx C, Sturt J, Skinner TC, Speight J. Diabetes Distress Among Adolescents with Type 1 Diabetes: a Systematic Review. Curr Diab Rep. 2016 Jan;16(1):9. PMID: 26748793. doi: 10.1007/s11892-015-0694-2.

12. Weissberg-Benchell J, Antisdel-Lomaglio J. Diabetes-specific emotional distress among adolescents: feasibility, reliability, and validity of the problem areas in diabetes-teen version. Pediatr Diabetes. 2011 Jun;12(4 Pt 1):341-4. PMID: 21443583. doi: 10.1111/j.1399-5448.2010.00720.x.
