# Supplementary material for: Real-Time Type 1 Diabetes Self-Management Decision-Making in Adolescents: Protocol for a Longitudinal Mixed Methods Study Using Text Messaging and Continuous Glucose Monitoring
Source: JMIR Res Protoc. 2026 Mar 4;15:e83218. doi: 10.2196/83218 (PMC12978980; doi:10.2196/83218)
Supplement: Multimedia Appendix 3 [file resprot-v15-e83218-s003.docx]

**Multimedia Appendix 3**. Real-time text message survey questions

Daily surveys (sent in the morning, early afternoon, late afternoon)

1. What have you been doing in the last few hours (at school/work, exercising, eating, etc.)? [Open-ended]
2. What’s going on with your diabetes right now? [Open]
3. Did you look at information from your diabetes devices in the last few hours [Y/N]
   1. (If No) Why not? [Open-ended]
   2. [Remaining questions if Q3 answered as Y]
4. What did you learn from your diabetes device(s)? [Open-ended]
5. How did you feel after looking at information from your diabetes device(s)? [Open-ended]
6. What did you do next? Or how did you use the information? [Open-ended]
7. Is there anything you want to tell us that happened in the last few hours? [Open-ended]

End-of-day reflection (sent in the evenings)

1. Overall, how did you feel about your diabetes today? [Open-ended]
2. Were your diabetes devices useful to you today? How?
3. What went well with your diabetes today? Or What was your “diabetes win” today? [Open-ended]
4. What problems, if any, did you encounter today? [Open-ended]
5. Use the daily graph in your CGM graph to review your blood sugars over the last 24 hours. Pick a time period you want to tell us more about (e.g., 8am to 10am). What time did you pick and why? [Open-ended]
6. Walk us through what you think impacted your blood glucose levels during this time of the day. [Open-ended]
7. Do you notice any patterns between your blood glucose levels in the past week? Tell us about it. [Open-ended]
8. How did taking care of your diabetes impact your day?
9. What else impacted how you took care of your diabetes today? [Open-ended]
10. Is there anything else you want to tell us that happened in the last few hours? [Open-ended]
