# Supplementary material for: Real-Time Type 1 Diabetes Self-Management Decision-Making in Adolescents: Protocol for a Longitudinal Mixed Methods Study Using Text Messaging and Continuous Glucose Monitoring
Source: JMIR Res Protoc. 2026 Mar 4;15:e83218. doi: 10.2196/83218 (PMC12978980; doi:10.2196/83218)
Supplement: Multimedia Appendix 4 [file resprot-v15-e83218-s004.docx]

**Closeout survey questions**

Thank you so much for participating in our study and sharing with us how you used your diabetes devices throughout the day. Now, we have some questions about what it was like to be a part of this study, by answering this survey.

1. What did you LIKE about being in the study? Why?
2. What did you DISLIKE about being in the study? Why?
3. What made it easy for you to participate in the study?
4. What got in the way of participating in the study?
5. How did you feel about being in the study for 30 days?
6. How did you feel about receiving text messages at 10am, 2pm, 6pm, and 10pm? Did these times generally work for you? Why or why not?
7. Is there anything else about your experience in this study you would like us to know?
