# Supplementary figures and images for: Real-Time Type 1 Diabetes Self-Management Decision-Making in Adolescents: Protocol for a Longitudinal Mixed Methods Study Using Text Messaging and Continuous Glucose Monitoring
Source: JMIR Res Protoc. 2026 Mar 4;15:e83218. doi: 10.2196/83218 (PMC12978980; doi:10.2196/83218)

**Multimedia Appendix 5.** Excerpt from example participant profile


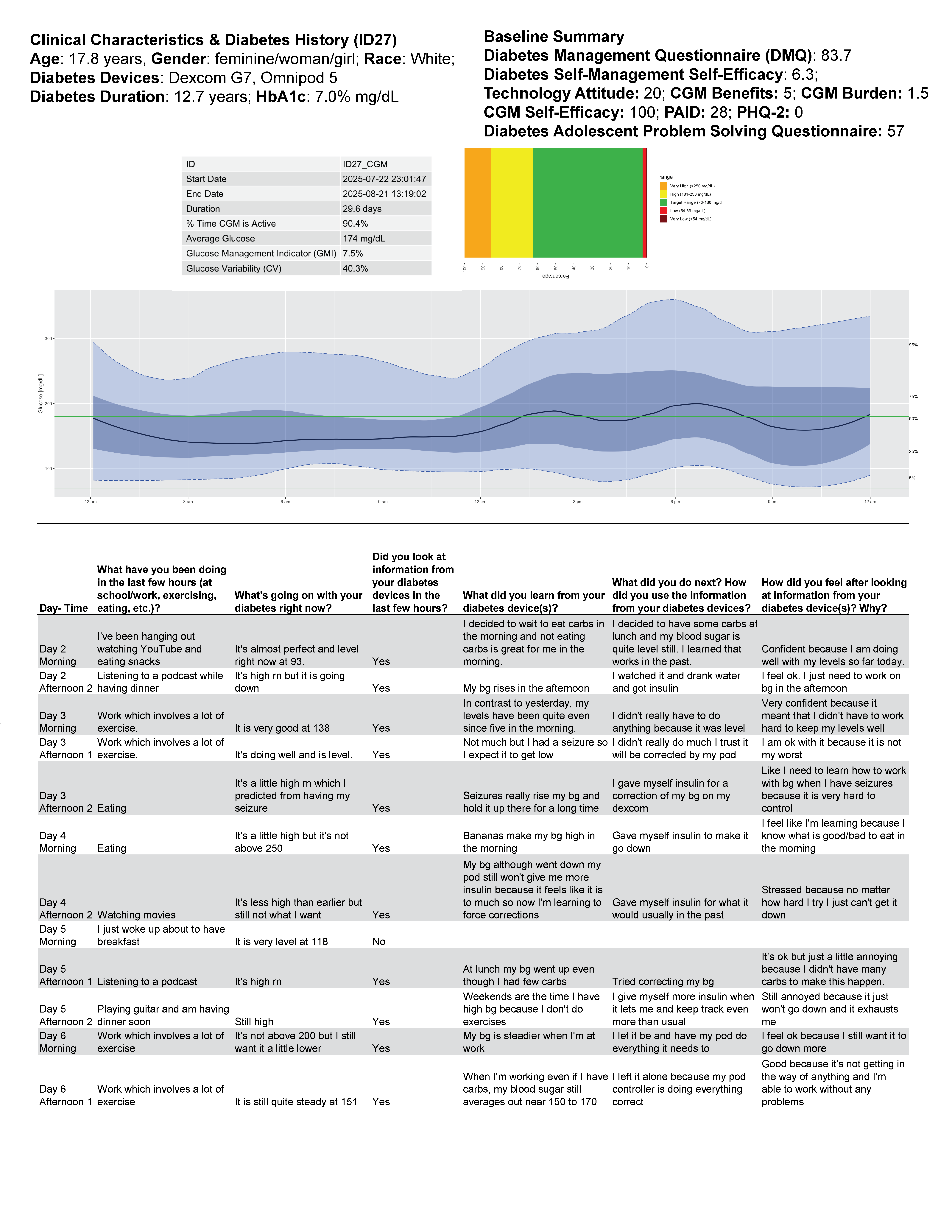

Supplement: Multimedia Appendix 5 [file resprot-v15-e83218-s005.docx]
